# TiO_2_ Nanotube Implants Modified with Silk Fibroin and Mesoporous Silica Nanocomposite Coatings Enable Efficient Drug Release to Promote Osteogenesis

**DOI:** 10.1021/acsami.5c03599

**Published:** 2025-04-28

**Authors:** Yanting Mu, Ming Li, Xiang Zhao, Chaihong Gong, Zhang Luo, Bing Li, Weiying Zhang, Xiaoxiao Ge, Su Chen, Jian Zhou

**Affiliations:** † Beijing Key Laboratory of Tooth Regeneration and Function Reconstruction, Beijing Stomatological Hospital, 12517 Capital Medical University, Beijing, 100071, China; ‡ China-America Institute of Neuroscience and Beijing Institute of Geriatrics, Xuanwu Hospital, 12517 Capital Medical University, Beijing, 100053, China; § Beijing Institute of Brain Disorders, 12517 Capital Medical University, Beijing, 100069, China; ∥ 74648 Shanxi Medical University School and Hospital of Stomatology, Taiyuan, 030001, China; ⊥ School of Life Science, Key Laboratory of Optoelectronic Chemical Materials and Devices of Ministry of Education, 74777 Jianghan University, Wuhan, 430056, China; # Beijing Laboratory of Oral Health, 12517 Capital Medical University, Beijing, 100069, China; ¶ Laboratory for Oral and General Health Integration and Translation, Beijing Tiantan Hospital, 12517 Capital Medical University, Beijing, 100070, China

**Keywords:** TiO_2_ nanotubes, osseointegration, drug delivery, coating, dental implant

## Abstract

Enhanced bone healing within 1 week after post-titanium (Ti) dental implant surgery especially contributes to the subsequent long-term osseointegration, and the commonly used drug-loaded TiO_2_ nanotubes (TNTs) can promote osteogenesis yet still face the challenge of burst drug release that makes it difficult to maintain long-term effective drug concentrations and good osseointegration. Here, we prepared a double drug loading/release system of silk fibroin/mesoporous silica nanoparticles (SF/MSN) nanocomposite coating modified TNTs (TAMA) with AZD2858 (Wnt/β-catenin pathway agonist for promoting osteogenesis) as the therapeutic drug, realizing a long-term stable drug release and better osteogenesis. The increased β-sheet content of SF reduced the degradation rate of the SF/MSN coating, thus avoiding the AZD2858 burst release. The adsorption of MSN maintained the effective drug concentration more than 1 week that was especially critical for early bone healing. Under the protection of SF/MSN coating, the TAMA implant showed a well-organized spatial release of AZD2858, well enabling the osteogenic differentiation and mineralization at cellular level for up to 21 days. Animal experiments further demonstrated that the slow release of AZD2858 in the TAMA implant effectively activated the Wnt/β-catenin pathway, enabling rapid bone healing in the early stage of implantation and finally achieving the best osseointegration efficacy. Thus, this study proposed an efficient strategy for developing high-performance dental implants via the construction of a biodegradable SF/MSN coating.

## Graphical abstract

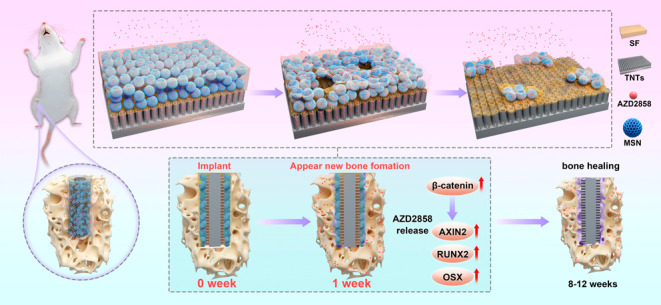

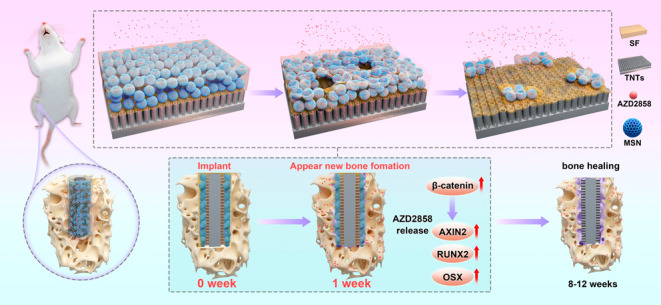

## 1 Introduction

Implant restoration is a conventional therapeutic approach for patients with dentition defects and edentulism. The remarkable biocompatibility and mechanical properties of titanium (Ti) enables its widespread use in clinical dental implants for achieving good osseointegration.^1,2^ After the placement of Ti implants, a small amount of woven bone could be observed on the implant surface as early as the seventh day. This “contact osteogenesis” represents the initial stage of osseointegration, with complete osseointegration achieved in 2–3 months.^3,4^ Furthermore, the earlier the formation of the new bone around the implant surface, the better the following osseointegration.^5^ However, optimally early bone healing is typically not realized with pure Ti implants owing to limitations associated with their biologically inert, which usually leads to implant loosening or even failure.^6,7^ Titanium dioxide (TiO_2_) nanotubes (TNTs) are obtained via the anodic oxidation of Ti and exhibit higher surface roughness and superior hydrophilicity than Ti; as a result, they greatly promote osteogenesis by improving the interaction between cells and the implants.^8^ Moreover, TNTs have a hollow structure, therefore serving as effective carriers for osteoinductive materials such as inorganic ions (calcium, strontium, and zinc) or biological molecules (dexamethasone, raloxifene, and bone morphogenetic protein-2).^9–11^ Local delivery of osteoinductive materials around the Ti implant minimizes the side effects associated with their systemic administration while concurrently allowing a reduction in drug doses and improving therapeutic efficacy.^12–19^ Despite various advantages, this system has the disadvantage of the burst release of drugs loaded in the TNTs within few hours of administration, resulting in short-term drug overload in the local tissues and difficulty in maintaining effective drug concentrations over a prolonged duration.^20,21^ Therefore, regulating the rate of drug release from TNTs and prolonging the release time are essential for optimizing the osseointegration of Ti implants.

Coating with natural degradable polymers has been reported to be an effective approach for regulating the rate of drug release.^22–25^ Silk fibroin (SF) extracted from silkworm cocoons comprises 18 amino acids and displays excellent biocompatibility and immunogenicity. Moreover, the mechanical properties and stability of SF can be regulated by altering its secondary structure. Thus, it is an ideal candidate for the development of biodegradable coatings.^26,27^ SF exists in the form of two crystalline structures, amorphous silk I and crystalline silk II; the presence of irregular coils, α-helices, and β-turn structures renders the former relatively unstable, while the β-sheet conformation of the latter endows it with a certain stability owing to the presence of hydrogen bonds and intermolecular forces.^27,28^ Adjusting the β-sheet content enables the optimization of the mechanical performance and biodegradable properties of SF.^29,30^ In addition, the amphiphilicity of SF facilitates drug loading.^31,32^ SF has been approved for clinical use in bone tissue repair by the United States Food and Drug Administration. Although coating Ti implants with SF to achieve controlled drug release has received considerable attention from the research community, in-depth studies are hitherto lacking and ideal therapeutic efficacy mediated by slow drug release remains to be attained.^33,34^ Mesoporous silica nanoparticles (MSNs) offer advantages of high specific surface area and pore volume, adjustable particle and pore sizes, presence of negative charges, and a considerable drug-carrying capacity.^35,36^ Furthermore, MSNs are biodegradable under *in vivo* conditions^37^ and are beneficial for maintaining normal growth and structural integrity of bones.^38^ Thus, integrating SF and MSNs for fabricating coatings to achieve controlled drug release has great potential for the development of functional Ti implants for optimizing osseointegration.

AZD2858, a selective inhibitor of glycogen synthase kinase-3 beta (GSK-3β), can directly activate the Wnt signaling pathway to promote bone mineralization and promote bone healing in rats.^39,40^ However, the commercially available AZD2858 exhibits poor solubility and is usually dissolved in dimethyl sulfoxide (DMSO) for subsequent systemic administration, resulting in a higher drug dose, prolonged duration of systemic circulation, and the inevitable side effects caused by DMSO.^41^ Herein, we describe the fabrication of an SF/MSN nanocomposite coating onto TNTs, resulting in the formation of a novel Ti implant (TAMA) capable of double-drug loading/release, with the drug AZD2858 encapsulated within both MSNs and TNTs. Compared with the requirements for systematic administration, utilizing the TAMA implant to locally deliver AZD2858 significantly reduced the drug dose and associated side effects. More importantly, adjusting the β-sheet content of SF effectively controlled the degradation rate of the SF/MSN coating, while the introduced MSNs increased the loading content of AZD2858 and contributed to slow drug release by absorbing AZD2858. In contrast to the observations with Ti implants, a prolonged release (over a week) of AZD2858 was attained with TAMA, with osteogenic differentiation and good osteogenic mineralization being achieved within 7–14 and 21 days, respectively. Furthermore, TAMA implantation in Sprague–Dawley (SD) rats exhibited the best osteogenesis performance, as evidenced by abundant new bone formation from 4 to 8 weeks after implantation. This biodegradable nanocomposite coating-based strategy is therefore suitable for prolonging the duration of local drug release around Ti implants and increasing their long-term stability (Figure 1).

**Figure 1:**
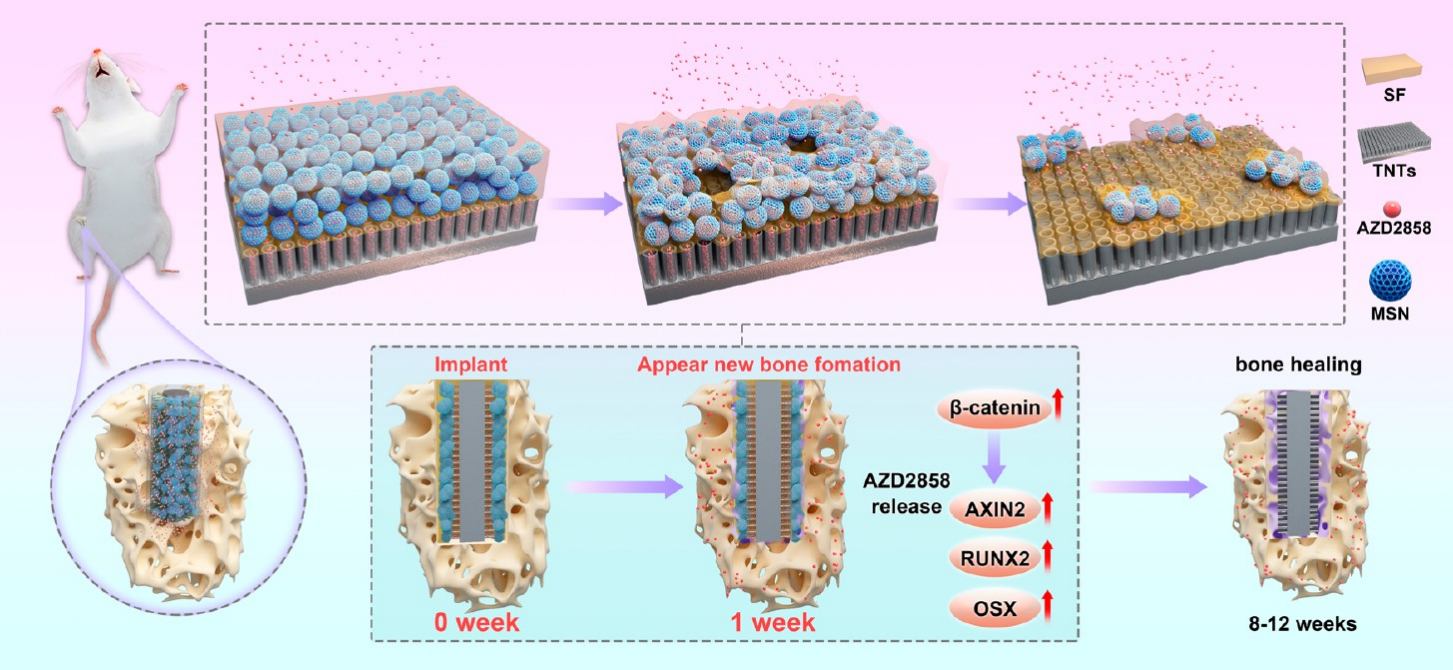
Loading the SF/MSN nanocomposite coating onto the surface of drug-loaded TNTs, which continuously release AZD2858 for more than 1 week around the implant to promote osseointegration.

## 2 Materials and Methods

### 2.1 Preparation of SF/MSN Nanocomposite Coating Modified TNTs

The TNTs were prepared as detailed previously.^42^ Squares (10 mm sides and 0.2 mm thickness) of Ti sheets (99.99%; Cuibolin Nonferrous Metal Industry Co., Ltd., China) were ultrasonically cleaned using acetone, ethanol, and deionized water, followed by anodic oxidation in ethylene glycol-based electrolyte solution containing 0.5% ammonium fluoride (w/v) and 10% (v/v) deionized water at 50 V for 15 min. The treated Ti samples were annealed in air at 500 °C for 2 h to obtain TNTs. This was followed by the addition of 20 μL of 2.5 μM AZD2858 solution (CAS No.: 486424-20-8, MedChemExpress, USA) on the TNTs and drying under negative pressure to obtain AZD2858-loaded TNTs (TNTs@AZD: TA).

The nanocomposite coatings were synthesized as follows. SF was first prepared from silkworm cocoons. The cocoons were boiled in a 0.02 mM sodium carbonate solution for 30 min for the removal of silk proteins (gelatin). The degummed SF was dissolved in a 9.3 M lithium bromide solution to obtain a uniform solution, which was dialyzed against deionized water for 48 h. The obtained SF solution was centrifuged, with subsequent analysis of the mass volume fraction of the final SF solution. MSNs were prepared as detailed previously,^35^ and 5 mg of MSNs was added to 2 mL of 50 μM AZD2858 solution. The mixture was stirred for 24 h to allow diffusion of AZD2858 molecules into the pores of MSNs, followed by centrifugation and washing with deionized water. The supernatant was discarded, and the precipitate was dried to obtain AZD2858-loaded MSNs (MSN@AZD: MSA). MSA was introduced into the above prepared SF solution (5% (w/v)), which was pretreated with ultrasonication (power 80 W, 30 s), resulting in the generation of an SF/MSA mixture with a concentration of 0.5 mg/mL. The surface of TA was coated with the SF/MSA solution (20 μL) and dried at 60 °C to yield TAMA (TA/SF/MSA). TAMA was further treated with ethanol (EtOH) to increase the β-sheet content of the nanocomposite coating, followed by drying at 37 °C. A similar procedure was employed for preparing different groups: TM (TNTs/SF/MSN), TMA (TNTs/SF/MSA), TAM (TA/SF/MSN), and TAS (TA/SF).

### 2.2 Characterization of Different Coating Modified TNTs

The morphology of the specimens modified with different coatings was analyzed by using a field-emission scanning electron microscope (FE-SEM; S4800, Hitachi, Japan). MSNs with and without drug loading were characterized by transmission electron microscopy (TEM; H-9000, Hitachi) under an operating voltage of 100 kV. The pore structure of MSNs was detected by using a physical adsorption analyzer (TriStar II 3020 3.02, Micromeritics, USA), with liquid nitrogen as the adsorption medium. A nanosize zeta potential analyzer (NanoBrook PALS 90plus, USA) was adopted to determine the zeta potential of MSNs before and after drug loading (n = 5). XPS analysis (Thermo Scientific K-Alpha, USA) was adopted to determine the valence states of elements in SF/MSN nanocomposite coating modified TNTs. Fourier transform infrared (FTIR) microspectroscopy (Nicolet iS5, USA) was employed to identify the characteristic groups of different coatings. The water contact angle of different coatings was measured using an optical contact angle measuring device (OCA 15pro, Dataphysics, Germany) (n = 5). The adhesion performance of the coatings was analyzed using a nanoindenter (Keysight G200, Keysight, USA); the surfaces of the specimens from each coating group were subjected to scratch tests using a three-sided pyramid indenter, with a maximum load of 100 mN, constant scratch speed of 30 μm/s, and scratch distance of 700 μm.

### 2.3 Degradation of SF/MSN Nanocomposite Coating and the AZD2858 Release

The drug loading rate of MSA was calculated in advance. MSA (0.5 mg/mL) in phosphate-buffered saline (PBS) was stirred at room temperature for 24 h. After centrifugation, the concentration of AZD2858 in the supernatant was quantified based on a standard curve of AZD2858 via liquid chromatography (E2695, Waters, USA). The rate of AZD2858 loading (*DL*) onto MSA was calculated using the following formula [Disp-formula eq1]:

$$DL\;\left(\%\right)=\frac{M_{A}}{M}\times 100\%$$ 1

where *M*_*A*_ and *M* denote the measured molar mass of AZD2858 in MSA and the mass of MSA, respectively.

The specimens modified with different coatings (TA, TAS, TMA, TAM, and TAMA) were placed in separate wells of a six-well plate. Following the addition of PBS (3 mL) to each well, the six-well plate was incubated at 37 °C for different durations (2, 4, 8, 12, 24, 48, 72, 96, 120, and 168 h). At the different time points, 500 μL of the incubated buffer was collected, which was replenished with an equal volume of fresh PBS. The collected incubated buffer was analyzed using liquid chromatography to determine the content of released AZD2858 in each drug-loaded group, and the percentage of the total released drug was calculated to determine the rate of drug release (n = 3).

The specimens modified with different coatings were incubated in a proteinase XIV solution (1 U/mL) in a 37 °C water bath. The coating specimens were removed every 2 days over a 14-day incubation period, rinsed with deionized water, freeze-dried, weighed, and then placed in a fresh proteinase XIV solution. The surface morphology of the coating specimens during the degradation process was evaluated using FE-SEM. The mass loss rate (*S*) of the samples was calculated using the following formula [Disp-formula eq2]:

$$S\;\left(\%\right)=\frac{R_{0}-R_{1}}{R_{0}}\times 100\%$$ 2

where *R*_*0*_ and *R*_*1*_ represent the initial mass of specimens and their mass after coating degradation, respectively (n = 5).

### 2.4 Evaluation of the *in Vitro* Osteogenic Ability of Different Specimens

Adhesion of cells on the different specimens was evaluated via FE-SEM analysis and fluorescence imaging of the cytoskeleton. The osteoblast cell line MC3T3-E1 (American Type Culture Collection, USA) was cultured in α-MEM medium containing 10% fetal bovine serum and 1% penicillin/streptomycin (Thermo Fisher Scientific) in a 5% CO_2_ atmosphere at 37 °C. MC3T3-E1 cells were seeded onto different coating specimens and incubated for 4 h. Then, the obtained specimens were rinsed twice with PBS and fixed overnight with 2.5% glutaraldehyde at 4 °C. The specimens were subsequently dehydrated using an ethanol gradient, followed by FE-SEM imaging for evaluating cell adhesion to the specimens. The obtained specimens were fixed overnight with 4% paraformaldehyde at 4 °C and then costained with fluorescein isothiocyanate (FITC)-labeled phalloidin (1:1000, 30 min; Abcam) and DAPI (5 min) for evaluating cell adhesion using confocal laser scanning microscopy (CLSM; LSM710, ZEISS, Germany).

The proliferative and osteogenic abilities of MC3T3-E1 cells on the different coating specimens were subsequently evaluated. Cell Counting Kit-8 assay (CCK-8, Dojindo, Japan) was employed for assessing the former. MC3T3-E1 cells were seeded onto the specimens in a 24-well plate and cultured for 1, 3, and 5 days. Subsequently, 10% CCK-8 solution was added to each well, followed by incubation for 2 h and measurement of absorbance at 450 nm (n = 5). The cell cycle was detected by flow cytometry using a cell cycle kit (Beyotime Biotechnology, Shanghai, China). Briefly, the cells were fixed overnight with 70% ethanol at 4 °C and stained with propidium iodide. The stained cells were analyzed with flow cytometry (n = 3). The osteogenic capacity of the specimens was evaluated based on alkaline phosphatase activity (ALP), calcium deposition staining, and reverse transcription quantitative real-time polymerase chain reaction (RT-qPCR). MC3T3-E1 cells were incubated with the specimens in a fresh osteogenic induction medium containing 100 nM dexamethasone (Solarbio, China), 10 mM β-glycerophosphate (Sigma-Aldrich Co., USA), and 50 mM l-ascorbic acid (Sigma-Aldrich Co.). After 7 and 14 days of incubation, total protein and ALP content were quantitatively evaluated using BCA protein assay (Beyotime, China) and ALP assay (Nanjing Jiancheng, China) kits, respectively. ALP activity was standardized with respect to the total protein content (n = 5). In addition, the cells were stained with a BCIP/NBT Kit (Beyotime, China) after 14 days of incubation, followed by imaging using a stereomicroscope (Olympus, Japan). For the analysis of calcium deposition, MC3T3-E1 cells were cultured for 21 days in a fresh osteogenic induction medium containing the specimens. Subsequently, MC3T3-E1 cells were incubated with a 2% alizarin red solution (Beyotime, China) at 4 °C for 30 min. Following thorough washing with deionized water, the cells were visualized and imaged by using a stereomicroscope. Alizarin red was dissolved in the solvent cetylpyridinium chloride (10% w/v), and absorbance was measured at 562 nm for quantifying the deposited calcium (n = 5). The expression of osteogenesis-related genes was analyzed via RT-qPCR. Following the coincubation of MC3T3-E1 cells with the specimens for 14 days, total RNA was extracted (TRIzol, Invitrogen, USA) and employed for reverse transcription using PrimeScript RT kit (TaKaRa, Japan). Commercially synthesized primers (Shenggong, China) were employed for amplifying mRNAs encoding β-catenin, AXIN2, and ALP; the mRNA encoding GAPDH was employed for normalization (n = 3). The primer sequences are listed in Table 1. The Western blotting was used to detect the expression level of Wnt/β-catenin signaling pathway-related proteins (β-catenin and RUNX2) and osteogenesis-related proteins (OCN). Briefly, total cellular protein of MC3T3-E1 cells was extracted using RIPA lysis buffer (Solarbio, China) after the coincubation with the specimens for 7 days. The extracted total protein was differentiated on a 20% sodium dodecyl sulfate polyacrylamide gel (GenScript, China). The proteins were then transferred to PVDF membranes (Millipore Corporation, Billerica, MA, USA). The membranes were blocked with 5% (w/v) nonfat milk for 1 h at room temperature and then incubated overnight at 4 °C with the following primary antibodies: β-catenin (66379-1-Ig, 1:1000, Proteintech), RUNX2 (CY5864, 1:1000, Abways), OCN (DF12303, 1:1000, Affinity BioSciences) and β-actin (20536-1-AP, 1:4000, Proteintech). Subsequently, the samples were then incubated with a secondary antibody (1:10,000, Abcom; 1:20,000, HUABIO) for 1 h at room temperature and visualized using an Immobilon Western Chemiluminescent HRP Substrate (Millipore Corporation, USA) (n = 3).

**Table 1:** Primers for RT-qPCR

Gene	Forward Primers (5′–3′)	Reverse Primers (5′–3′)
β-catenin	ATGGAGC­CGGACAG­AAAAGC	CTTGCCACT­CAGGGA­AGGA
AXIN2	GTCTCTA­CCTCATTT­CCCGAGAAC	CGAGATCAG­CTCAGC­TGCAA
ALP	TGCCCTG­AAACTCC­AAAAGC	CTTCACGCC­ACACAA­GTAGG
GAPDH	ATGGGTG­TGAACCA­CGAGA	CAGGGATGA­TGTTCT­GGGCA

### 2.5 Evaluation of the *in Vivo* Osteogenic Ability of Different Specimens

Implants of 1 mm diameter and 8 mm height modified with different coatings (Ti, TAM, and TAMA) were prepared as described previously. Six-week-old male SD rats were randomly assigned to three groups, and each received one implant in the femur (n = 5). At 4- and 8-weeks postimplantation, the bone samples containing the implants were harvested for assessing new bone formation. In addition, blood samples were collected for hematological analysis and evaluation of liver and kidney functions, and the major organs (heart, liver, spleen, lungs, and kidneys) were collected for hematoxylin and eosin (H&E) staining. The femurs of SD rats were subjected to high-resolution microtomography (micro-CT; Skyscan, Bruker, Germany) with a spatial resolution of 18 μm. The region of interest was defined as a cylindrical region of 2 mm diameter, which extended downward from the epiphyseal growth plate to a length of 4 mm along the implant. CT-Analyzer was employed for data reconstruction to obtain three-dimensional (3D) images. Four bone parameters, including new bone volume over total bone volume (BV/TV), mean trabecular thickness (Tb.Th), trabecular number (Tb.N), and trabecular separation (Tb.Sp), were evaluated to assess osseointegration. The femur samples were fixed with 4% paraformaldehyde, subjected to hard tissue sectioning, and stained with methylene blue and acid fuchsin. Simultaneously, the femur samples were decalcified using 10% ethylenediamine­tetraacetic acid solution and subjected to H&E staining.

Furthermore, the femur sections of experimental SD rats were subjected to immunofluorescence analysis to demonstrate the therapeutic effects of implants modified with different coatings, activation of the Wnt/β-catenin signaling pathway, and promotion of osteogenesis. RUNX2, OSX, β-catenin, and AXIN2 were employed as indicators to evaluate the activation of the Wnt/β-catenin signaling pathway by the implants modified with different coatings, while ALP, collagen-I, OPN, and OCN were employed as osteogenesis-associated indicators. For immunofluorescence staining, the femur sections were incubated overnight with primary antibodies at 4 °C and, subsequently, with secondary antibodies for an hour. The nuclei were stained with DAPI for 5 min, and the sections were imaged using CLSM. ImageJ software was employed for precisely quantifying the positive-staining signals. The following antibodies were employed for immunofluorescence staining at a dilution of 1:100: RUNX2 (AF5186, Affinity BioSciences), OSX (DF7731, Affinity BioSciences), β-catenin (66379-1-Ig, Proteintech), ALP (DF6225, Affinity BioSciences), AXIN2 (DF6978, Affinity BioSciences), OPN (AF0227, Affinity BioSciences), and OCN (DF12303, Affinity BioSciences). Collagen-I antibody (67288-1-Ig, Proteintech) was used at a dilution of 1:200. FITC-conjugated goat antirabbit IgG, iFluor 594-conjugated goat antimouse IgG, and iFluor 647-conjugated goat antirabbit IgG (all obtained from HUABIO) were employed as secondary antibodies at dilutions of 1:500.

All experimental protocols were approved by the Animal Ethics Committee of Beijing Stomatological Hospital, Capital Medical University, China. The care and use of all rats were in accordance with the National Guidelines for the Care and Use of Laboratory Animals.

### 2.6 Statistical Analysis

All data were expressed as the mean ± standard deviation using SPSS 26.0. Analysis of variance was employed to calculate P-values in comparisons involving multiple groups with Tukey’s posthoc test as the multiple comparisons correction. Statistically significant differences (*P* < 0.05) were indicated by different lowercase letters at certain instances and with *, **, and ***, denoting *P* < 0.05, *P* < 0.01, and *P* < 0.001, respectively, at other instances.

## 3 Results and Discussion

The SF/MSN nanocomposite coating modified TNTs were prepared as detailed in the schematic depicted in Figure 2a. MSA was mixed homogeneously with ultrasound-treated SF and added dropwise onto the surface of TA; this TA was dried at 60 °C and treated with EtOH to obtain TAMA (TA/SF/MSA). The change in morphology during the preparation of TAMA was evaluated by using FE-SEM (Figure 2b). Pure Ti exhibited a relatively smooth surface with a few visible scratches, while TA exhibited a uniform array of nanotubes of approximately 100 nm diameter and 1-μm height. TAS displayed an approximately 10-μm-thick layer over TA, thereby efficiently circumventing the exposure of TA; this was particularly beneficial for mitigating the burst release of loaded drugs. With TAMA, the SF/MSN nanocomposite coating resulted in spherical mesoporous particles of approximately 200 nm diameter inside the SF coating, which corresponded well with the size of MSNs observed in the TEM images (Figure 2b and Figure 2c). The evaluation of nitrogen adsorption onto MSNs revealed a typical adsorption–desorption isotherm of mesoporous materials with a narrow and relatively homogeneous pore distribution (Figure 2d and Figure 2e), which is highly conducive for drug loading. In contrast to the characteristics of MSNs, MSA exhibited a relatively rough surface, indicating the successful loading of AZD2858 (Figure S1). Zeta potential evaluation revealed a reduced negative charge of MSA compared to that of MSNs owing to AZD2858 loading (Figure S2). The mesoporous morphology and negative charges of MSNs promoted drug loading and the adsorption of the amino-containing drug AZD2858 based on the principle of microsphere drug loading (drug embedding and electrostatic adsorption),^43^ thus reducing the rate of AZD2858 release to a certain extent. Clinically, dental implants are subjected to insertion torque during surgical placement, underscoring the importance of interfacial adhesion between coatings and titanium substrates. Scratch tests were performed to characterize the bonding ability of the SF/MSN nanocomposite coating on TA. The detection principle was mainly based on the transition of scratch curves from smooth to fluctuating, indicating interface fracture (coating delamination); the load corresponding to this point was defined as the critical load value. The TAMA implant (TA modified with SF/MSA coating) failed to exhibit any obvious fluctuations in the scratch curves, indicating no significant peeling or fracture in the coating on the implant; this is critical for countering certain torsional forces associated with the surgical placement of the implant (Figure 2f). XPS analysis was adopted to determine the valence states of elements in SF/MSN nanocomposite coating modified TNTs. The obtained result clearly showed that TAS, TM, TMA, TAM and TAMA all have obvious O 1s, N 1s and C 1s peaks, due to the presence of SF, MSN and possibly adsorptive water (Figure S3). It should be noted that the Si 2p peak was not observed. It is speculated that the content of MSNs in the SF coating is relatively low in comparison to SF, and the MSNs are almost entirely encased inside the SF. Hence, the covered silicon signal is difficult to detect. Furthermore, the water contact angles were measured to demonstrate the change in hydrophilicity of the different specimens (Figure 2g and Figure S4). In contrast to hydrophobic Ti, TNTs exhibited significantly increased hydrophilicity owing to the anodization process. Moreover, the presence of polar functional groups in SF, including amino (−NH_2_) and carboxyl (−COOH) groups, considerably increased the hydrophilicity of the Ti implant.^44^ Thus, the SF coating increased the water contact angle of TAS to approximately 68.9°. Moreover, MSNs led to synergistic effects on porosity^45^ to further improve the hydrophilicity of the specimens TM, TMA, TAM, and TAMA with nanocomposite coating; additionally, the contact angles were reduced to nearly 58°, resulting in a surface with better hydrophilicity than TAS. Thus, the good hydrophilicity and bonding ability of the SF/MSN coating significantly improved the potential clinical efficacy of TAMA implants.

**Figure 2:**
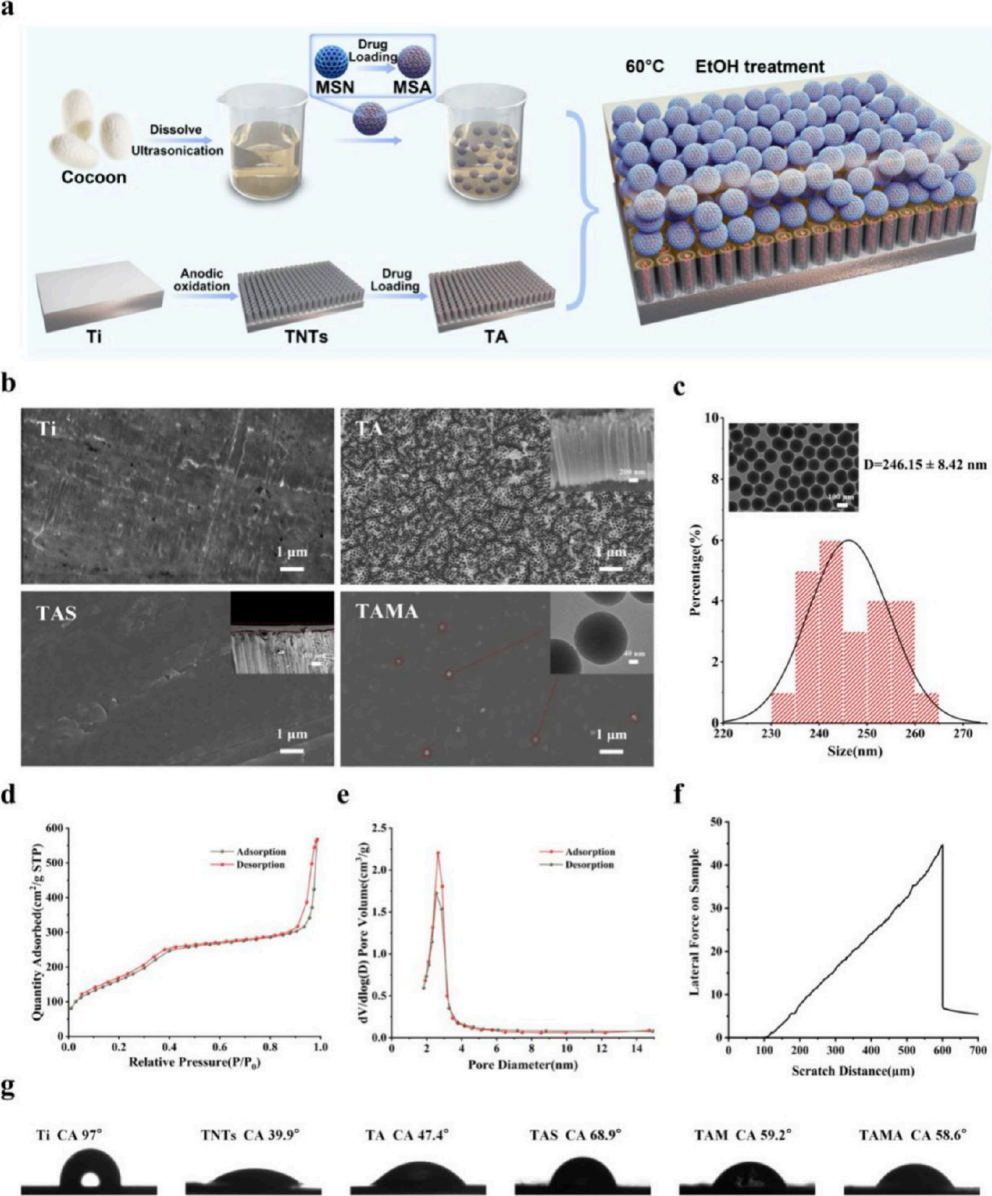
Characterization of different specimens. (a) Loading the SF/MSN nanocomposite coating on the surface of AZD2858-loaded TNTs. (b) FE-SEM and TEM images of surface and cross-sectional morphologies of different specimens. (c) The pore size distribution analysis of MSN. (d) The particle size distribution analysis of MSN. (e) The nitrogen adsorption–desorption isotherm of MSN. (f) Nanoscratch results for TAMA. (g) Water contact angle of different specimens.

SF is the main component in the construction of different coatings, and the β-sheet content in the secondary structure of SF is closely associated with its degradation. Under equilibrium conditions, a higher β-sheet content in SF leads to increased structural stability and slower coating degradation, which is beneficial for slowing down the drug release rate.^46^ Thus, the secondary structure of the different coatings was evaluated using FTIR spectroscopy. The characteristic peaks in the amide I region (1600–1700 cm^–1^), which are associated with the secondary structure of SF, shifted from 1640 to 1622 cm^–1^ following temperature elevation and ultrasound as well as EtOH treatments compared to those in SF coating without any treatment (Figure 3a, Figure 3b, and Figure S5 and Table S1), indicating a significantly high formation of β-sheets. Furthermore, a quantitative analysis of the characteristic peaks in the amide I region clearly showed that the β-sheet content in all of the coatings was considerably higher than that in untreated SF, and the addition of MSNs or MSA did not significantly impact the secondary structure of SF. In addition, the degradation behavior of SF/MSN coatings was explored by determining the weight change in TAMA and other groups following incubation with proteinase XIV for different durations. The untreated SF coating underwent complete degradation within 2 days, while the coatings with the β-sheet structure required up to 12 days (Figure 3c). The increased β-sheet content of SF substantially improved the stability of the SF/MSN coating and simultaneously decreased its degradation rate. The extent of the AZD2858 loading in TAMA was subsequently analyzed. As per the calculations, the *DL* value for MSA was 0.28%. Then, the different specimens loaded with AZD2858 were incubated in PBS for different durations, and the released AZD2858 was determined; the distinguished drug release behaviors are shown in Figure 3d and Figure 3e. For the uncoated group (TA), the burst release of the drug within 12 h was observed. TAS prolonged drug release up to 96 h, with a slower release rate in the initial 12 h. The above phenomenon can mainly be attributed to the abundance of −NH_2_ and −COOH groups in SF molecules, which facilitates the adsorption of drug molecules onto SF,^30,46^ as well as to the slow degradation of SF coatings, which reduces the drug release rate especially in the early stages. The mesoporous morphology and negative charges of MSNs especially promoted the absorption of the amino-containing AZD2858 molecules; TAM therefore exhibited extended and efficient drug release for up to 168 h. However, up to 80% of the drug in the TAM group was released within 120 h. TAMA exhibited the slowest release rate and the best sustained drug release behavior, as evidenced by the retention of 21.7% of loaded AZD2858 even after 168 h. This can primarily be attributed to increased AZD2858 loading content due to the presence of MSNs in the coating, the readsorption of the released drug onto MSNs, and the slow degradation rate of SF. Moreover, the relationship between AZD2858 release and SF/MSN nanocomposite coating degradation in the TAMA implant was explored using FE-SEM (Figure 3f). The surface of the nanocomposite coating began to disintegrate, and MSA (AZD2858-loaded MSN) simultaneously emerged on day 1. With increasing degradation time, the nanocomposite coating exhibited an uneven surface, and MSA in the deeper layers was gradually exposed. After 7 days, a large area of TA was observed. The stepwise degradation of the SF/MSN coating is believed to indirectly indicate the slow release rate of AZD2858 from the TAMA implant.

**Figure 3:**
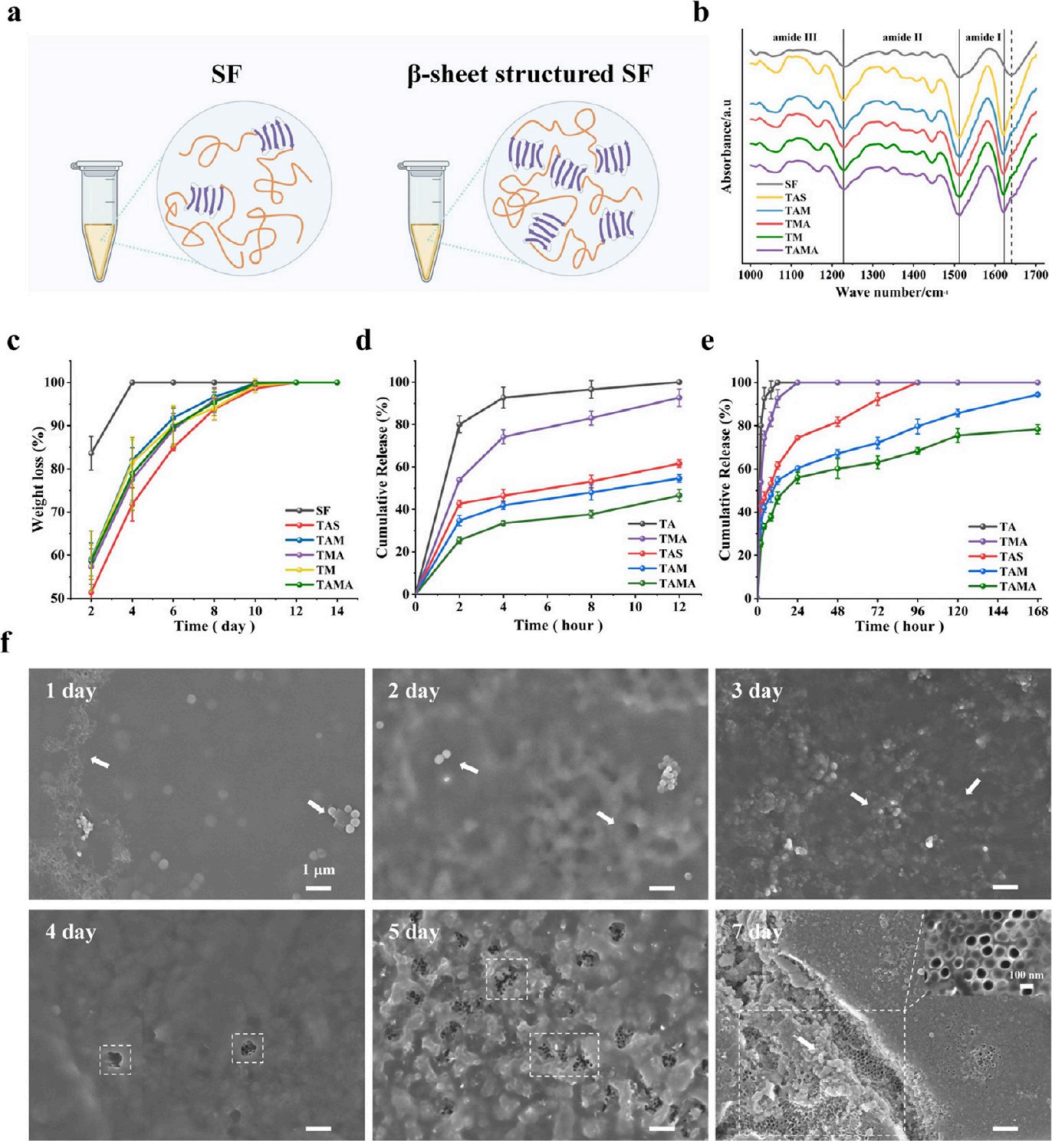
Drug release and degradation behavior of the coatings. (a,b) Coatings secondary structure analysis by FTIR spectroscopy. (c,d) The drug release of AZD2858 from different specimens. (e,f) The degradation behavior of the coating of TAMA. Figure 3a: Created in BioRender. Mu, Y. (2025) https://BioRender.com/opi22ox.

Supported by the above results, the osteogenesis ability of the TAMA implant was evaluated by using cell-based experiments. As previously reported, the physical properties of materials can directly influence cell adhesion and spreading.^47–49^ The results of the FE-SEM analysis clearly revealed that MC3T3-E1 cells did not fully spread on the surface of Ti and exhibited fewer pseudopodia (Figure 4a). By contrast, better spreading of MC3T3-E1 cells was observed on the surface of TNTs, TA, and TAS, with the cells appearing more flattened and with significantly increased numbers of pseudopodia. The good hydrophilicity of the groups with SF/MSN nanocomposite coating (including TM, TMA, TAM, and TAMA) enabled considerably better dispersion of the cells, tighter bonding, and greater numbers of pseudopodia. In addition, fluorescence staining for cytoskeletal elements demonstrated that the best cell adhesion was achieved with TAMA (Figure S6). AZD2858 regulates the growth of bone marrow mesenchymal stem cells by modulating the Wnt/β-catenin signaling pathway, while simultaneously promoting cell proliferation and osteogenic differentiation.^50–52^ In this work, the AZD2858 release rate was found to be the key factor affecting osteogenic differentiation. The proliferation of MC3T3-E1 cells following exposure to the different specimens was evaluated by using the CCK-8 assay, as depicted in Figure 4b. The proliferative activities of cells in all the groups increased over time, and the optimal cell proliferation rate was attained with TAMA, mainly due to its optimized drug release behavior. The MC3T3-E1 cell proliferation cycle on the different specimens was tested through the flow cytometry analysis, as shown in Figure S7. The obtained results displayed that the number of cells in the S phase and G2/M phase after coincubated with TAMA greatly increased in comparison to Ti, well indicating that TAMA promoted the replication and division of DNA. The results were consistent with the previous CCK-8 results. ALP activity is an important indicator of both early- and midstage osteogenic differentiation, and calcium deposition was used for evaluating late-stage osteogenic differentiation. The ALP activities following the incubation of MC3T3-E1 cells with the different specimens for 7 and 14 days are shown in Figure 4c and Figure 4d. The ALP activity of TA was higher than that of Ti and TNTs owing to AZD2858 loading but lower than that of TAS. The ALP activity of TAM was higher than that of TAS, which can primarily be attributed to the controlled release of AZD2858. As expected, TAMA exhibited drug release over the longest period and the highest ALP activity (Figure 4c). Furthermore, the results of ALP staining were consistent with those of quantitative ALP analysis, with TAMA exhibiting the highest level of expression of ALP (Figure 4d). Late-stage osteogenic differentiation was evaluated via alizarin red staining to assess calcium deposition in MC3T3-E1 cells treated with the different specimens (Figure 4e and Figure 4f). TAMA exhibited the most obvious calcium deposition compared with the other groups, indicating that late-stage osteogenic differentiation was optimally facilitated by TAMA. RT-qPCR was performed to detect the expression levels of genes associated with osteogenic differentiation, including β-catenin, AXIN2, and ALP. Elevated expression levels of these three genes were observed in all the AZD2858-loaded groups (Figure 4g). A comparison of the different coatings loaded with AZD2858 revealed that the longest duration of drug release, greatest extent of cell proliferation/differentiation, and significantly enhanced expression of β-catenin and AXIN2 were exhibited by the TAMA implant, followed by TAM and TAS. The Western blotting was used to detect the expression level of Wnt/β-catenin signaling pathway-related proteins (β-catenin and RUNX2) and osteogenesis-related proteins (OCN). After MC3T3-E1 cells cocultured on different specimens for 7 days, TAMA exhibited the obviously highest expression of β-catenin, RUNX2 and OCN in comparison to the other groups, well indicative of the activation of Wnt/β-catenin signaling pathway caused by the continuous release of AZD2858 and the followed improved osseointegration performance (Figure 4h and Figure 4i). Moreover, the TM implant (TNTs/SF/MSN) without drug loading demonstrated good cell proliferation and osteogenic differentiation compared to that observed with the Ti implant; this observation is in line with previously published results that SF and silicon ions promote cell metabolism and enhance new bone formation.^53^

**Figure 4:**
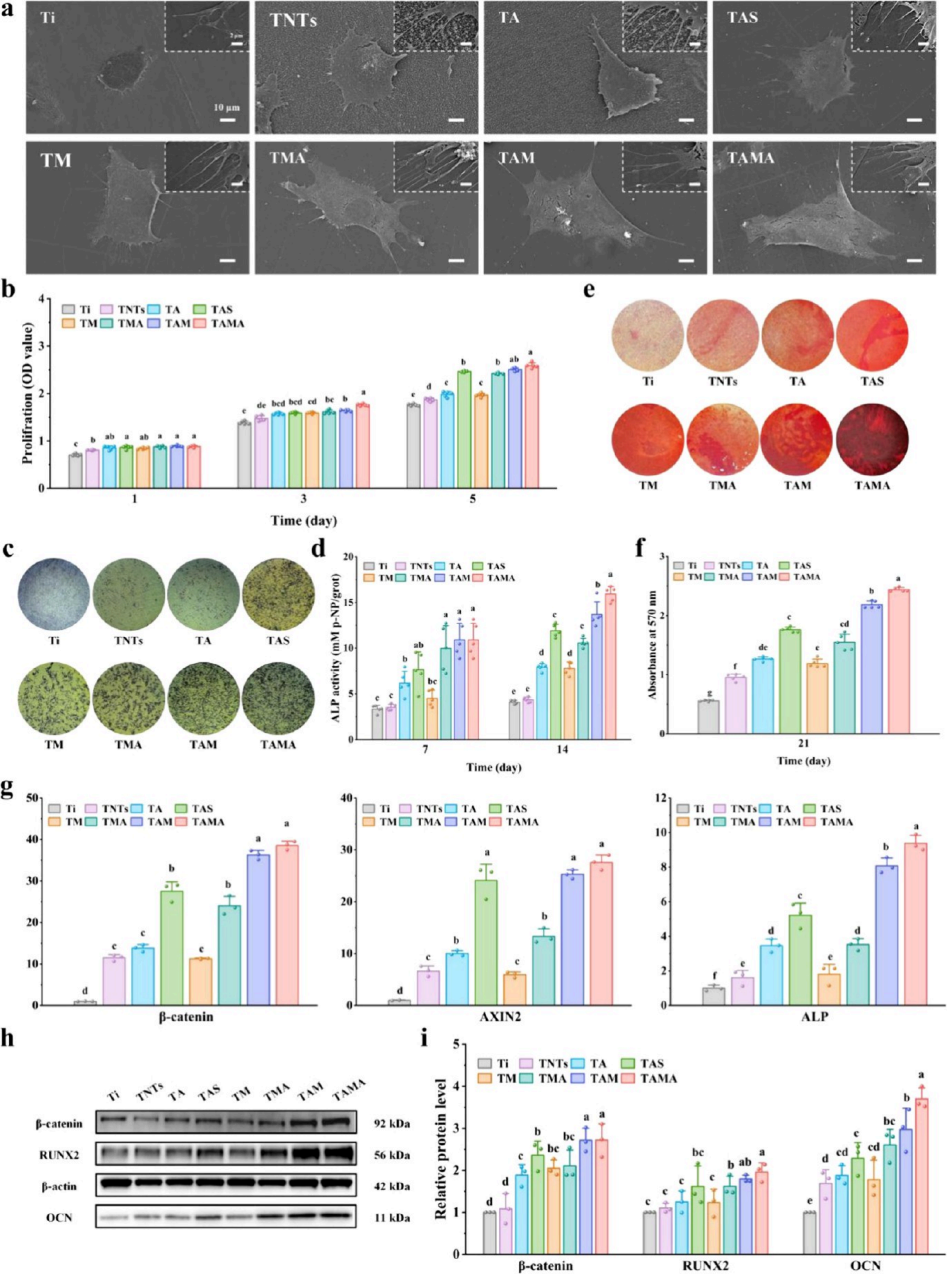
*In vitro* biocompatible and osteogenic assessment of MC3T3-E1 cells. (a) Surface morphology (FE-SEM) of cells adhered on different specimens after incubation for 4 h. (b) Cell proliferation ability evaluated through CCK-8 for 1, 3, and 5 days. (c and d) ALP quantitative and staining results of cells incubated on the different specimens for 7 and 14 days of osteogenic induction. (e and f) Alizarin red staining and quantitative results of the cells seeded on different specimens at 21 days of osteogenic induction. (g) mRNA levels of osteogenic differentiation-related genes (β-catenin, AXIN2 and ALP) in cells cultured on the different specimens at 14 days after seeding in osteogenic medium. (h) Western blotting assay of β-catenin, RUNX2 and OCN protein expression of MC3T3-E1 cells cultured on different specimens for 7 days, and (i) relative density quantification was normalized to β-actin.

The results of the *in vitro* cell-based experiments indicate that the introduction of an SF/MSN nanocomposite coating enables drug loading/release with greater efficacy and facilitates superior osteogenic differentiation. TAM and TAMA were employed to evaluate the *in vivo* osteogenesis ability of the SF/MSN nanocomposite coating, and Ti served as the control. Blood samples and the main organs (heart, liver, spleen, lungs, and kidneys) were obtained from 6-week-old male SD rats with Ti, TAM, and TAMA implants to evaluate key indicators in blood and conduct histological analysis. After 4 and 8 weeks of implantation, no significant differences were observed in the results of blood biochemistry and blood routine analyses in the TAM and TAMA groups compared with the Ti group (Figure S8). Furthermore, histological analysis revealed that TAM and TAMA implants did not cause any abnormality in the main organs (Figure S9). These results indicate the adequate safety of the degradation products of the SF/MSN coating (amino acids and silicon ions), highlighting the excellent potential of the TAMA implant for dental implantation.

The experimental rats were subjected to micro-CT analysis to evaluate the integration of the different implants (Ti, TAM, and TAMA) with the surrounding bone, as shown in Figure 5a and Figure 5b. Following implantation, the Ti implant did not support good bone healing, resulting in a larger extent of bone defect; by contrast, the TAM implant exhibited better osseointegration, owing to the controlled drug release associated with the SF/MSN nanocomposite coating. The TAMA implant exhibited AZD2858 release over the longest period and the best osseointegration, as reflected by the smallest bone defect area. 3D micro-CT reconstruction imaging clearly showed that TAMA supported the highest degree of surrounding bone formation. A simultaneous quantification of the micro-CT data demonstrated the peri-implant new bone quality and indirectly reflected the metabolic status of the bone, with BV/TV, Tb.N, Tb.Th, and Th.Sp serving as the evaluation indices (Figure 5c). The increase in the BV/TV value indicates that anabolic rather than catabolic processes are dominant in the bone, resulting in an increase in bone mass. Furthermore, active anabolism in the trabecular bone is reflected in the increased values of Tb.N and Tb.Th. Following implantation, the TAMA implant exhibited good BV/TV and Tb.Th values, which were nearly two times higher than those of the Ti implant. Moreover, the Tb.N values for the TAMA implant were significantly higher than those for the Ti implant. Significant differences in Th.Sp values were not observed among the three groups, indicating that the prepared implants did not cause apparent separation of the trabeculae. These results demonstrate that the TAMA implant promotes anabolic processes in the surrounding bone tissue, resulting in the greatest formation of new bone and trabeculae, which reflects its excellent bone healing capacity.

**Figure 5:**
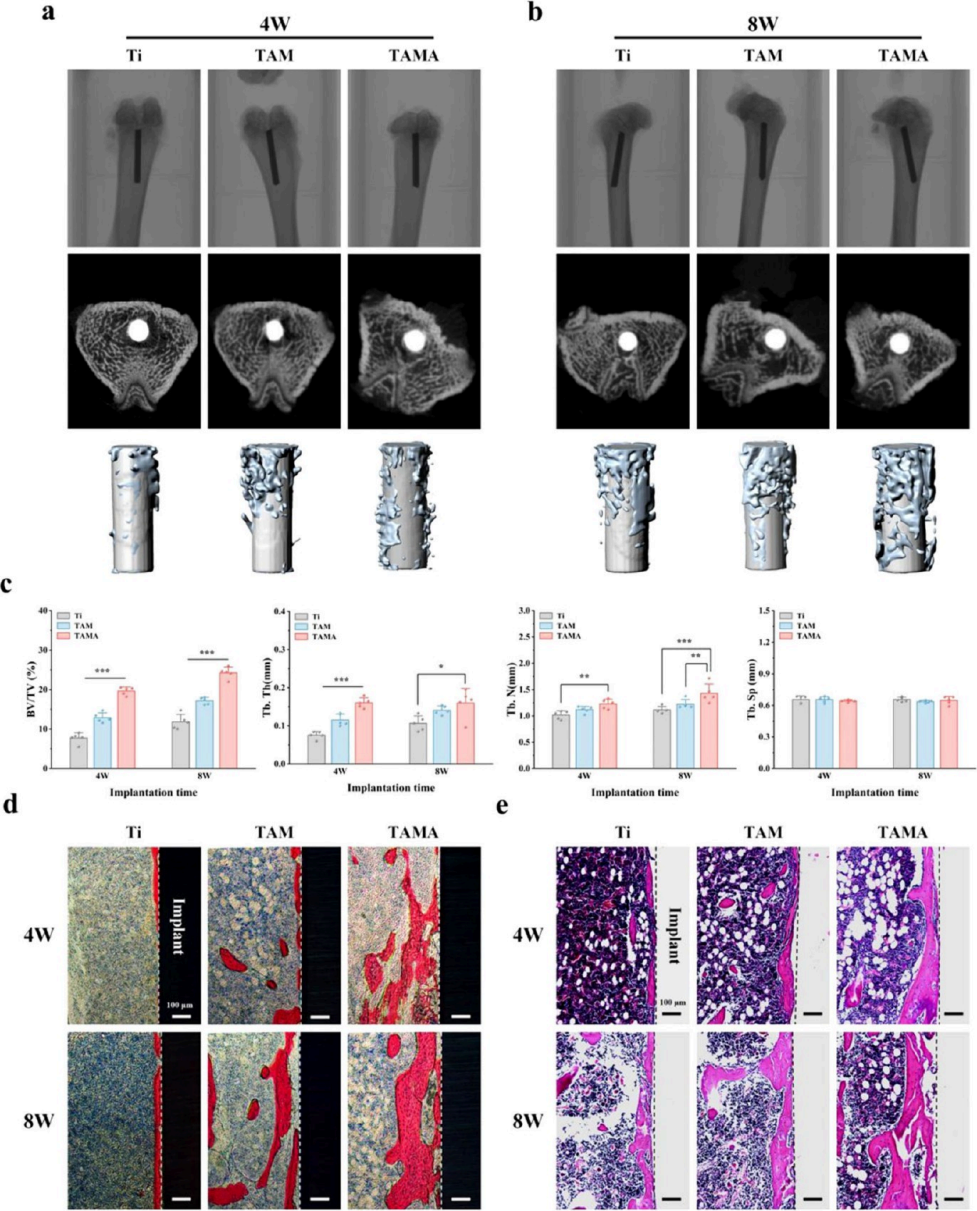
*In vivo* osteogenic assessment. (a and b) Representative 3D-reconstructed micro-CT images of the bone around each group of implants after 4 and 8 weeks. (c) Quantitative statistics of BV/TV, Tb.Th, Tb.N and Tb.Sp according to the micro-CT images after 4 and 8 weeks. (d and e) Histological analysis of peri-implant new bone formation by methylene blue/acid fuchsin and H&E staining after 4 and 8 weeks (*P < 0.05, **P < 0.01, ***P < 0.001).

The integration of surrounding bone with the implants was evaluated using methylene blue and acid fuchsin staining (Figure 5d); the newly formed bone surrounding the implants was stained red, while the osteoblasts were stained blue. The results revealed that 4 weeks after implantation, only limited new bone formation was observed around the Ti implant, which increased after 8 weeks but remained restricted to the surface of the implant. By contrast, a greater extent of new bone formation was observed at 4 weeks postimplantation of the TAM implant compared to that of the pure Ti implant, which became more pronounced at 8 weeks. As expected, optimal new bone formation was observed at 4 and 8 weeks postimplantation of the TAMA implant, which is attributable to the fact that the SF/MSN coating effectively prolonged the duration of AZD2858 action at the interface between the implant and bone tissue, thereby promoting osseointegration. The results of H&E staining were consistent with the above results (Figure 5e), with optimal osseointegration attained with the TAMA implant.

Immunofluorescence analysis was performed to investigate the effects of slow release of AZD2858 from the Ti implants with different coatings on the activation of the Wnt/β-catenin signaling pathway and osseointegration (Figure 6). AZD2858 functions as an inhibitor of GSK-3β and a negative regulator of this signaling pathway, preventing the degradation of β-catenin while promoting its translocation into the nucleus.^54,55^ The nuclear accumulation of β-catenin activates the expression of downstream target genes such as those encoding RUNX2, OSX, and AXIN2, which ultimately promote osteoblast differentiation and bone mineralization.^56^ At 8 weeks after implantation, the TAMA and TAM implants increased the expression levels of RUNX2, OSX, β-catenin, and AXIN2 compared to the Ti implant, indicating the activation of the Wnt/β-catenin signaling pathway by AZD2858. Benefiting from the SF/MSN coating, which supports a dual system of AZD2858 loading and release, TAMA supported the sustained activation of the Wnt/β-catenin signaling pathway with a greater efficacy than TAM, with higher expression levels of RUNX2, OSX, β-catenin, and AXIN2. Subsequently, the expression of osseointegration-related proteins was evaluated. In contrast to the bare Ti implant, both TAM and TAMA supported high expression levels of ALP, collagen-I, OPN, and OCN, suggesting that good osseointegration is attained with these two implants. The apparently higher expression levels of osteogenesis-related proteins engendered by the TAMA implants strongly demonstrate that their dual drug-loading system and slow drug release behavior support better activation of the Wnt/β-catenin signaling pathway and improve the efficiency of osseointegration.

**Figure 6:**
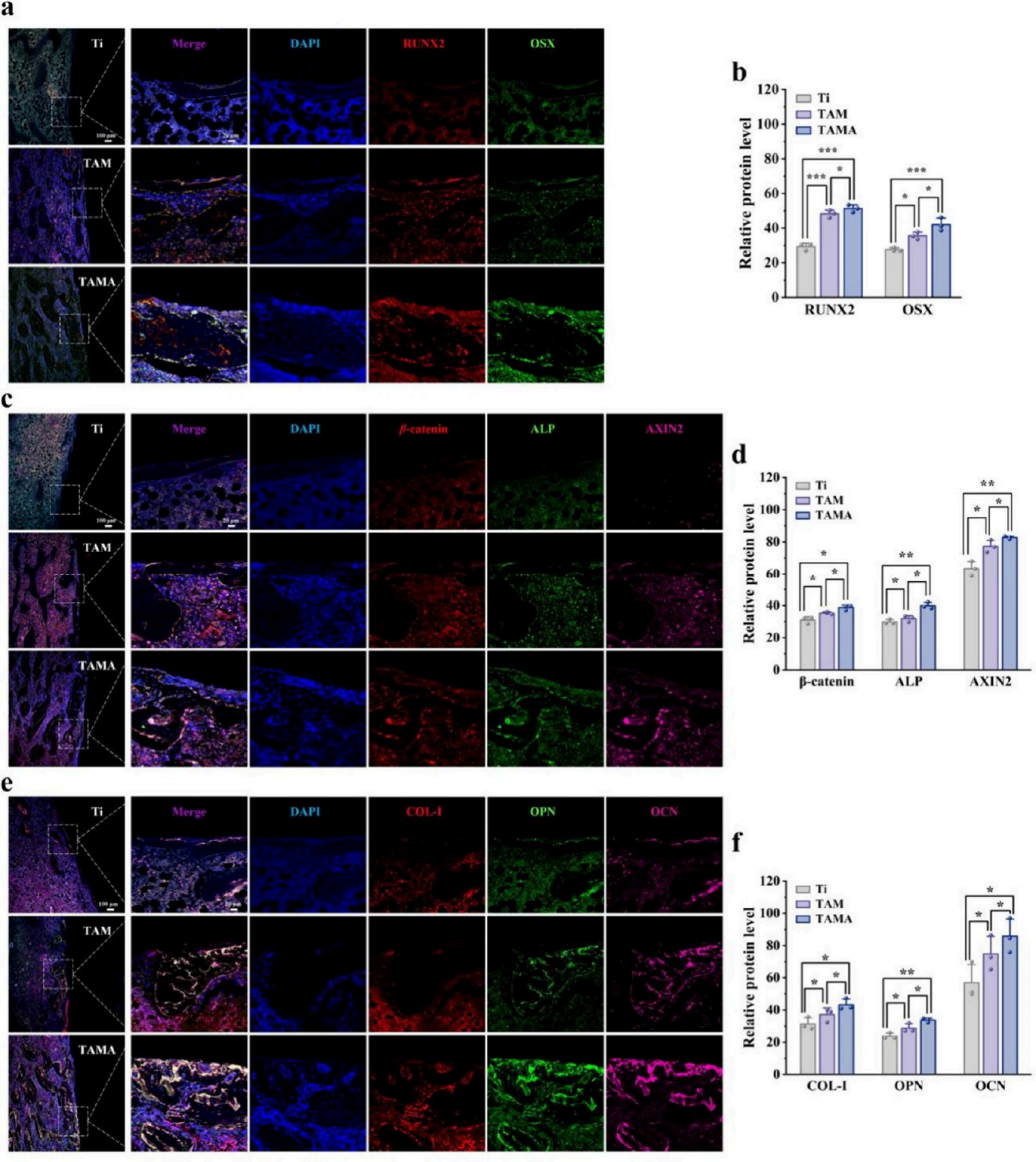
TAMA implants accelerate osseointegration by rapid activation of Wnt/β-catenin pathway. (a) and (b) Representative images of immunofluorescence costaining RUNX2 (red) and OSX (green) of the bone tissue in each group of implants after 8 weeks and the quantitative analysis of fluorescence intensity. (c and d) Representative images of immunofluorescence costaining β-catenin (red), ALP (green) and AXIN2 (pink) of the bone tissue in each group of implants after 8 weeks and the quantitative analysis of fluorescence intensity. (e and f) Representative images of immunofluorescence costaining Collagen-I (red), OPN (green) and OCN (pink) of the bone tissue in each group of implants after 8 weeks and the quantitative analysis of fluorescence intensity. *P < 0.05, **P < 0.01, ***P < 0.001.

## 4 Conclusion

In summary, we describe the fabrication of an SF/MSN nanocomposite coating with remarkable biocompatibility and biodegradability as well as the development of the TAMA implant that can support dual drug loading and release, which is achieved by loading AZD2858 onto both TNTs and the SF/MSN coating. A localized and slow release of drug AZD2858 around the dental implant was efficiently achieved with the TAMA implant, effectively circumventing the burst release of the drug, which usually occurs with the commonly used Ti implants. Moreover, the TAMA implant supported stable and prolonged release of the drug AZD2858 for up to 7 days, which proved to be especially beneficial for ensuring continued activation of the Wnt/β-catenin signaling pathway and promoting early bone healing. Both *in vitro* and *in vivo* experiments confirmed that the TAMA implant supported optimal osseointegration. Thus, the current study presents a good strategy for achieving the regulated (slow) and localized release of drugs from dental implants, greatly contributing to innovations in implant technology.

## Supplementary Material



## Notes

FTIR analysis of the secondary structure of SF coatings; Zeta potential analysis of MSN and MSA; XPS analysis of the SF/MSN nanocomposite coating modified TNTs; water contact angles of different specimens; cytoskeletal elements fluorescence staining of MC3T3-E1 cells; flow cytometry analyais of MC3T3-E1 cells proliferation cycle; blood indicators analysis of experimental rats; H&E staining of the major organs. (PDF)
