# Supplementary material for: TiO2 Nanotube Implants Modified with Silk Fibroin and Mesoporous Silica Nanocomposite Coatings Enable Efficient Drug Release to Promote Osteogenesis
Source: ACS Appl Mater Interfaces. 2025 Apr 28;17(21):30600–12. doi: 10.1021/acsami.5c03599 (PMC12123568; doi:10.1021/acsami.5c03599)
Supplement: Supplementary file 1 [file am5c03599_si_001.pdf]

Supporting Information

TiO_2 nanotube implants modified with silk fibroin and mesoporous silica nanocomposite coatings enable efficient drug release to promote osteogenesis

Yanting Mu [a, 1], Ming Li [b, 1], Xiang Zhao [a, c, d], Chaihong Gong [c, e], Zhang Luo [c, e], Bing Li [d], Weiying Zhang [e], Xiaoxiao Ge [c, *], Su Chen [a, *], Jian Zhou [a, f, g, *]

[a] *Beijing Key Laboratory of Tooth Regeneration and Function Reconstruction, Beijing Stomatological Hospital, Capital Medical University, Beijing, 100050, China.*

[b] *China-America Institute of Neuroscience and Beijing Institute of Geriatrics, Xuanwu Hospital, Capital Medical University; Beijing, 100053, China.*

[c] *Beijing Institute Brain Disorders, Capital Medical University, Beijing, 100069, China.*

[d] *Shanxi Medical University School and Hospital of Stomatology, Taiyuan, 030001, China.*

[e] *School of Life Science, Key Laboratory of Optoelectronic Chemical Materials and Devices of Ministry of Education, Jianghan University, Wuhan, 430056, China.*

[f] *Beijing Laboratory of Oral Health, Capital Medical University, Beijing, 100069, China.*

[g] *Laboratory for Oral and General Health Integration and Translation, Beijing Tiantan Hospital, Capital Medical University, Beijing, 100070, China.*

[1] These authors contributed equally to this paper.

* Corresponding author: xiaoxiaoge@ccmu.edu.cn (Xiaoxiao Ge), 13910164776@163.com (Su Chen), zhoujian@ccmu.edu.cn (Jian Zhou).

Table S1 The FTIR analysis of the secondary structure of SF coatings

	Wave number (cm^{-1})	SF	TAS	TM	TMA	TAM	TAMA
***β*-sheets**	1610-1635, 1695-1700	29.9%	42.7%	42.5%	42.6%	41.6%	41.1%
**random**	1635-1645	22.1%	15%	16.4%	16.8%	15.6%	15.7%
***α*-helices**	1658-1664	9.6%	6.0%	8.7%	9%	8.7%	8.9%
**turns**	1647-1654	14.8%	10%	8.9%	8.4%	9.3%	9.8%

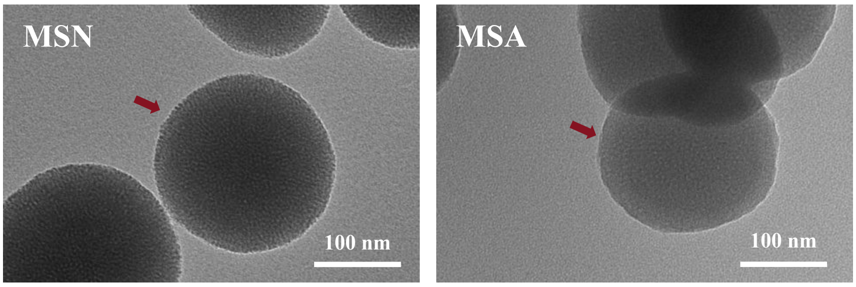

Figure S1. TEM images of MSN and MSA.

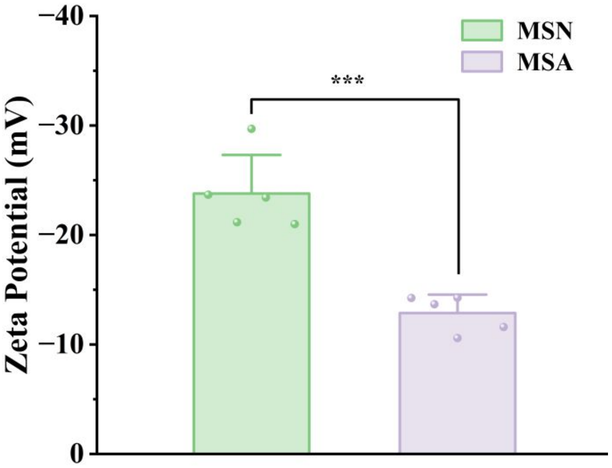

Figure S2. The Zeta potential analysis of MSN and MSA.

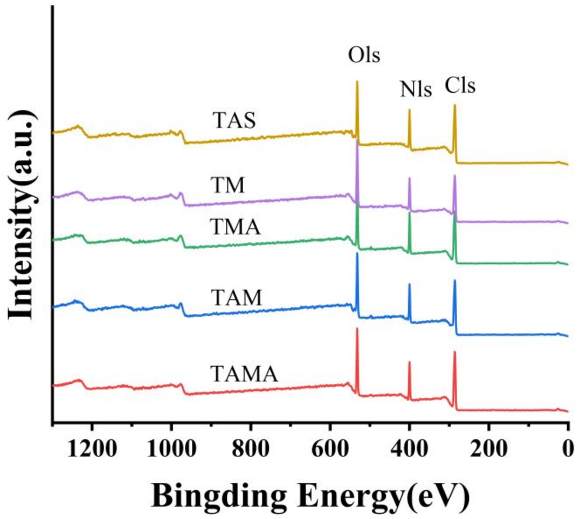

Figure S3. The XPS analysis of the SF/MSN nanocomposite coating modified TNTs.

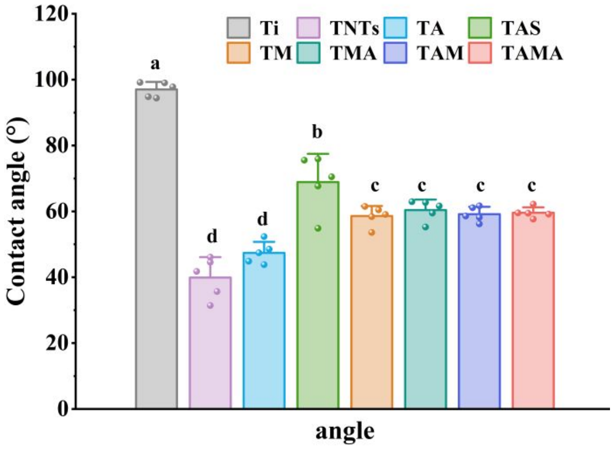

Figure S4. Water contact angles of different specimens.

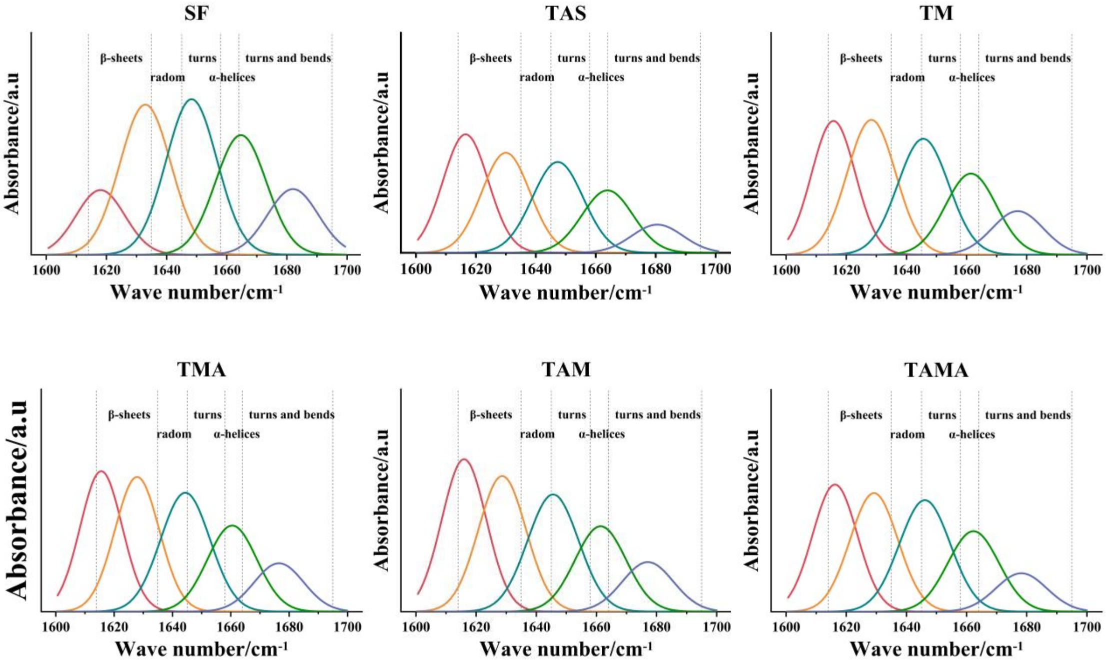

Figure S5. The FTIR analysis of different coatings.

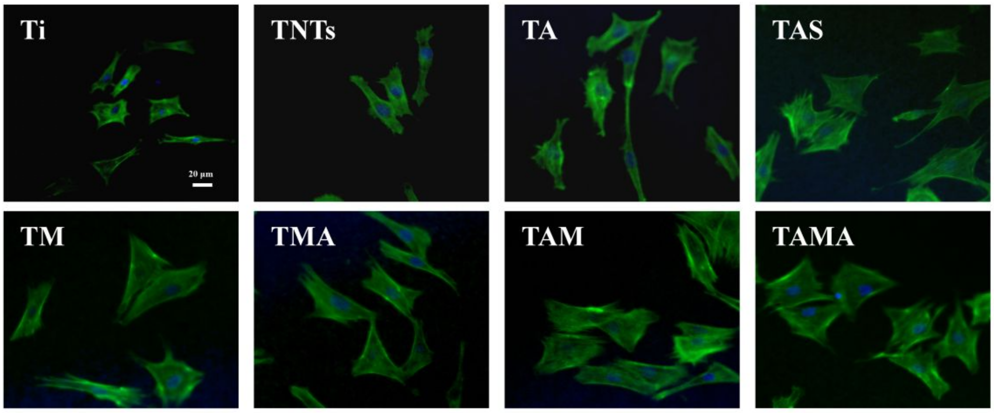

Figure S6. The staining images of cytoskeletal actin fibers (green) and nuclei (blue) for MC3T3-E1 cells on different specimens after incubation for 4 hours.

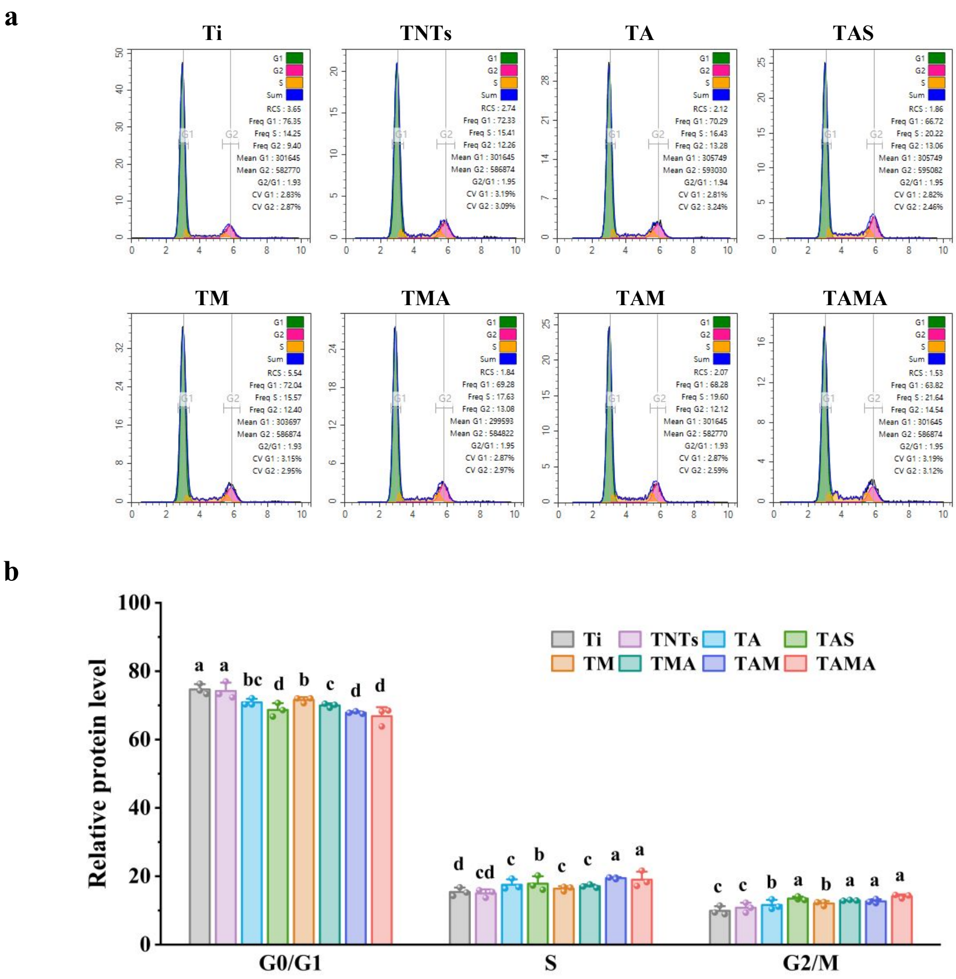

Figure S7. (a) The flow cytometry analyais of MC3T3-E1 cells proliferation cycle on the different specimens, and (b) the related bar scatter plots.

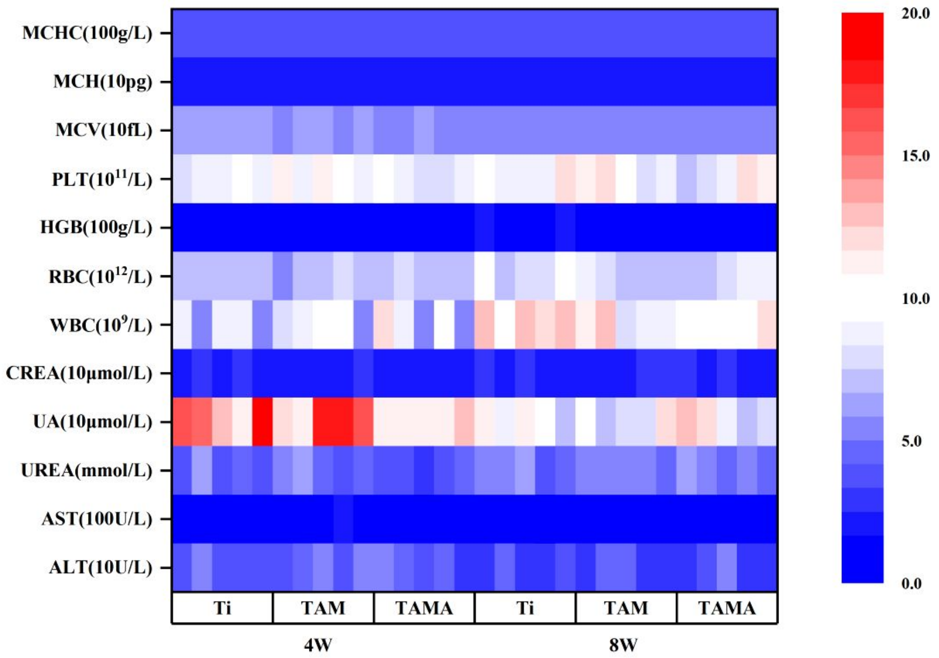

Figure S8. The blood indicators analysis of experimental rats after the implantation of different implants, MCHC: mean corpusular hemoglobin concerntration, MCH: mean corpuscular hemoglobin, MCV: mean corpuscular volume, PLT: platelets, HGB: hemoglobin, RBC: red blood cells, WBC: white blood cells, CREA: creatinine, UA: uric acid, AST: aspertate aminotransferase, ALT: aminotransferase.

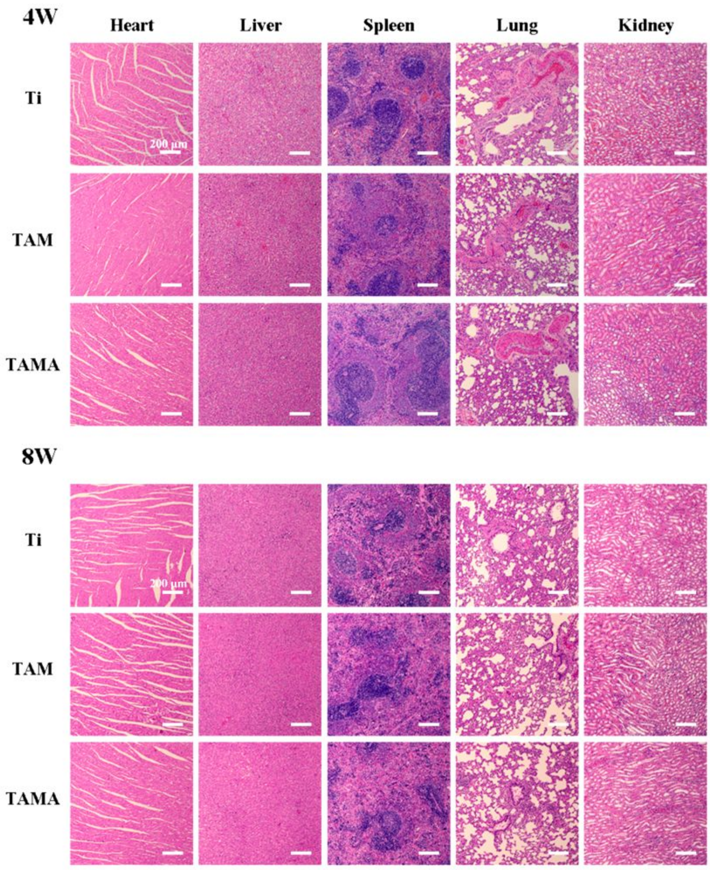

Figure S9. H&E staining of the major organs (heart, liver, spleen, lung and kidney) of experimental rats treated with different implants for 4 weeks and 8 weeks.
